# Diagnostic Accuracy of the HemoCue Hb 301, STAT-Site M^Hgb^ and URIT-12 Point-of-Care Hemoglobin Meters in a Central Laboratory and a Community Based Clinic in Durban, South Africa

**DOI:** 10.1371/journal.pone.0152184

**Published:** 2016-04-05

**Authors:** Manjeetha Jaggernath, Rumallen Naicker, Savathree Madurai, Mark A. Brockman, Thumbi Ndung’u, Huub C. Gelderblom

**Affiliations:** 1 HIV Pathogenesis Programme, Doris Duke Medical Research Institute, University of KwaZulu-Natal, Durban, South Africa; 2 Global Clinical and Viral Laboratory, Durban, South Africa; 3 KwaZulu-Natal Research Institute for Tuberculosis and HIV, University of KwaZulu-Natal, Durban, South Africa; 4 International AIDS Vaccine Initiative (IAVI), New York, NY, United States of America; 5 Simon Fraser University, Burnaby, BC, Canada; 6 British Columbia Centre for Excellence in HIV/AIDS, Vancouver, BC, Canada; 7 Ragon Institute of MGH, MIT and Harvard, Charlestown, MA, United States of America; 8 Max Planck Institute for Infection Biology, Berlin, Germany; Gentofte University Hospital, DENMARK

## Abstract

In South Africa, various point-of-care hemoglobin meters are used. However, the regulatory framework for approval, implementation and oversight of use of point-of-care hemoglobin meters is suboptimal. We assessed the diagnostic accuracy of the HemoCue Hb 301, STAT-Site M^Hgb^ and URIT-12 point-of-care hemoglobin meters, compared to a central laboratory based reference assay, in a central laboratory and a community based clinic in Durban, South Africa. Differences in performance of the point-of-care assays, compared to the reference assay, were more pronounced in the community based clinic. Results were reasonable for the HemoCue Hb 301, but poor for the STAT-Site M^Hgb^ and the URIT-12. Poor test performance of point-of-care hemoglobin meters, and inadequate evaluations and oversight in South Africa, leads to suboptimal clinical care and clinical research, and increased costs. There is a need for proper evaluation and quality assurance of point-of-care tests, the results of which should be made widely available to key stakeholders.

## Introduction

Anemia is common in sub-Saharan Africa [1], and a frequent reason for exclusion from clinical studies [2]. During screening procedures for enrollment in clinical studies, hemoglobin levels are usually assessed using portable point-of-care hemoglobin meters with blood obtained by finger prick. Portable point-of-care assays can give results within minutes, allowing rapid clinical decision-making, during the encounter with the patient [3]. But point-of-care test results must be accurate. Inaccurate point-of-care hemoglobin meters that misdiagnose anemia may lead to (i) false exclusion from, and inclusion in, clinical studies, and (ii) compromise clinical care.

In South Africa there is suboptimal government regulation on the approval and use of point-of-care assays. During screening procedures for a clinical study we observed discrepant results between a point-of-care hemoglobin meter and a laboratory-based testing platform in a certified central laboratory. We therefore performed a prospective study to assess the diagnostic accuracy of three point-of-care hemoglobin meters in (i) a certified central laboratory and (ii) in a community based clinic, compared to a central laboratory reference assay.

## Materials and Methods

### Study design

We determined the diagnostic accuracy of three point-of-care hemoglobin meters in a 2-phase study. In phase 1, the point-of-care tests were performed in a central laboratory (Global Clinical and Viral Laboratory in Amanzimtoti), compared to a laboratory reference standard. In phase 2, the point-of-care tests were performed in a community based clinic (iThembalabantu clinic in the Umlazi township) and the reference test was performed in the central laboratory, using a sample obtained at the same time as the point-of-care testing. The study was approved by the institutional review board of the University of KwaZulu-Natal. For phase 1 we used anonymized routine blood samples. For phase 2, written informed consent was obtained from each patient in English or isiZulu (the predominant local language).

### Patient selection

Patients were eligible for inclusion in phase 2 of the study if they met the following criteria: Age 18 years or older, present for clinical care or HIV screening at one of our research clinics or during outreach activities in the greater Durban area, willing to provide written informed consent, and willing to undergo study procedure.

### Specimen collection

During phase 1 of the study, we used anonymized EDTA whole blood samples submitted to the central laboratory for routine hematology testing. During phase 2 of the study, we used blood obtained by finger prick for the three point-of-care tests performed in the community based clinic, and we drew EDTA whole blood samples for the reference hemoglobin test in the central laboratory. The distance between the community based clinic and the central laboratory was 15km. Samples were transported from the community based clinic to the central laboratory once or twice a day.

### Hemoglobin assessments

We evaluated three point-of-care hemoglobin meters: URIT-12 (URIT Medical Electronic Group, Guilin, Guangxi, China [4]), STAT-Site M^Hgb^ (Stanbio Laboratory, Boerne, TX, USA [5]), and HemoCue Hb301 (HemoCue AB, Ängelholm, Sweden [6]). We compared the results from the point-of-care hemoglobin meters to those obtained on an automated hematology analyzer (Sysmex XS-1000i, Sysmex, Kobe, Hyogo, Japan [7]) as a reference hemoglobin meter in a South African National Accreditation System (SANAS) certified laboratory. The Sysmex XS-1000i was used according to the manufacturer’s recommendations. Calibration was performed annually. QC was performed daily with high, medium and low controls. The total allowable error for hemoglobin was 4.19%. External quality assurance of the laboratory was performed every 4 months. All point-of-care hemoglobin meters were used according to the manufacturers’ recommendations. During phase 1, one technician conducted all tests. During phase 2, one nurse conducted all point-of-care tests in the community based clinic, and one technician subsequently conducted all reference laboratory tests in the central laboratory; the technician was blinded to the point-of-care test results. All tests were performed as single measurements. The point-of-care tests were conducted in random order. Anemia was defined according to World Health Organization criteria as hemoglobin < 12 g/dl for women, and < 13 g/dl for men [8].

### Statistical analysis

We used GraphPad Prism and Microsoft Excel for graphical representation and statistical analysis. We assessed the accuracy, as measured by the mean difference (bias) and 95% limits of agreement, of the HemoCue 301, URIT-12 and STAT-Site M^Hgb^ point-of-care hemoglobin meters compared to the laboratory hemoglobin test as a reference using the Bland-Altman method [9]. We assessed reproducibility (inter-assay variation) during phase 1 by repeat testing a subset of samples with all assays.

## Results and Discussion

### Results phase 1, central laboratory

During phase 1 of the study, we tested samples from 60 patients in a central laboratory. We performed phase 1 of the study from August 19 to 27, 2013. We performed all four tests on individual samples on the same day.

**Fig 1:**
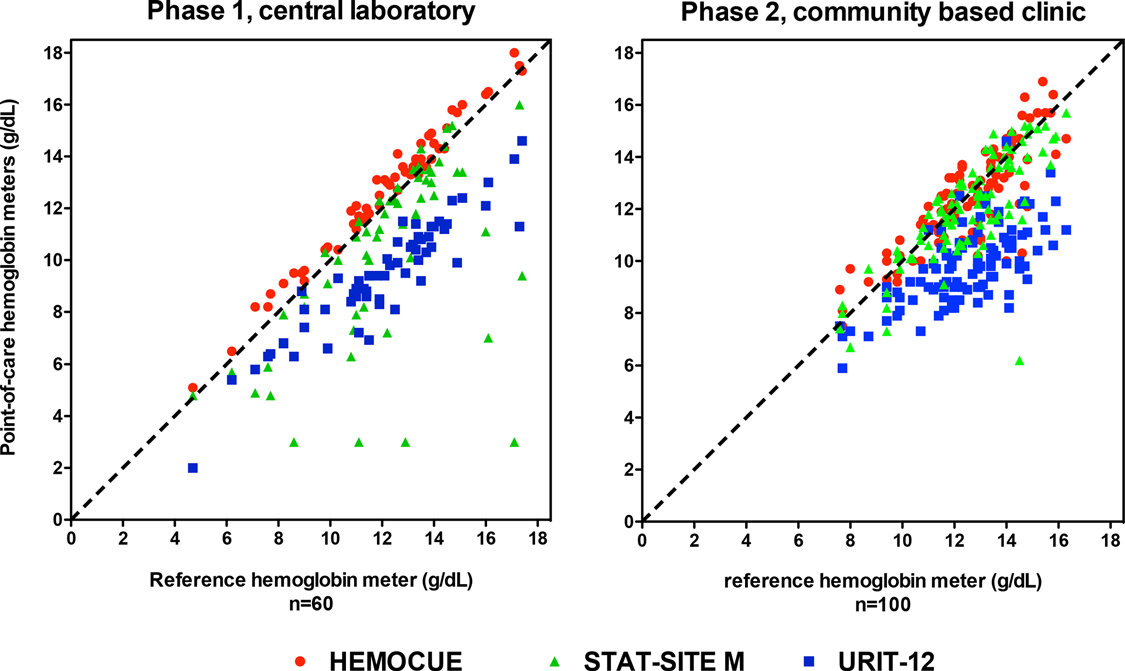
Correlation of the 3 point-of-care assays with values within the dynamic range of the reference Hemoglobin meter in 60 samples in a central laboratory (phase 1), and 100 samples in a community based clinical setting (phase 2). In phase 1 of the study the STAT-Site had a high failure rate. It seems that this was related to uneven migration of the sample through the sample strip, resulting in the sample migration time exceeding the maximum time specified by the manufacturer.

**Fig 2:**
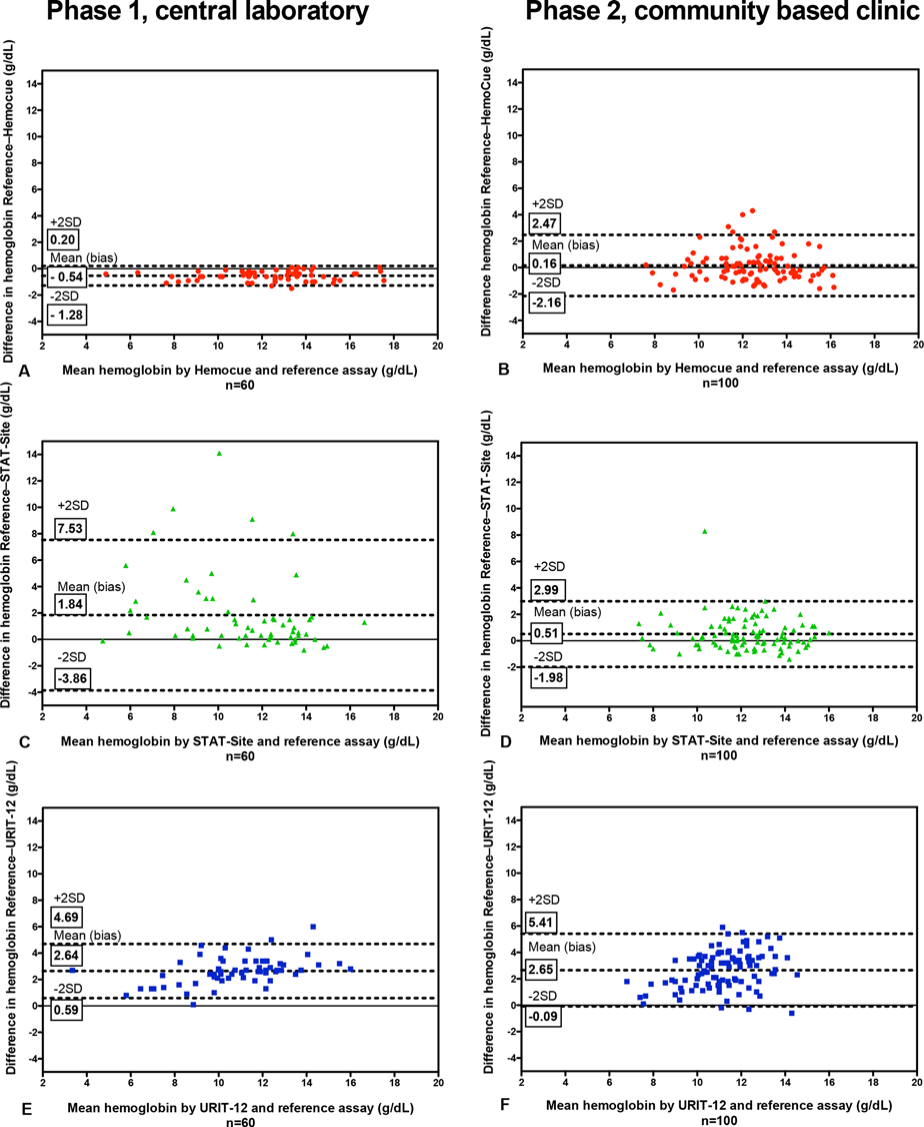
Bland-Altman plots comparing the reference laboratory test with the 3 point-of-care assays in phase 1 (left column) and phase 2 (right column). In phase 1 of the study the STAT-Site had a high failure rate. It seems that this was related to uneven migration of the sample through the sample strip, resulting in the sample migration time exceeding the maximum time specified by the manufacturer.

As depicted in Figs 1 and 2, compared to the Sysmex XS-1000i laboratory reference, results were most accurate for the HemoCue (bias –0.54, limits of agreement –1.28 to 0.20 g/dL, CV <1%), less accurate for the STAT-Site M^Hgb^ (bias 1.84, limits of agreement –3.86 to 7.53 g/dL, CV = 30.4%) and least accurate for the URIT-12 (bias 2.64, limits of agreement 0.59 to 4.69 g/dL, CV <5%). The sensitivity and specificity of the point-of-care assays to detect or exclude anemia in the central laboratory is summarized in S1 Table, and S1 Fig.

### Results phase 2, community based clinic

During phase 2 of the study, we tested samples from 100 patients (63 women and 37 men) in a community based clinic using three point-of-care devices with blood obtained by finger prick, and compared them with the laboratory reference using a venous EDTA blood sample drawn at the time of point-of-care testing. The median duration between venous blood draw and sample receipt at the central laboratory was 3 hours and 21 minutes (range 38 minutes–8 hours and 25 minutes). The median duration between venous blood draw and test result at the central laboratory was 5 hours and 9 minutes (range 1 hour and 15 minutes–9 hours and 37 minutes). We performed phase 2 of the study from February 10 to 17, 2014. The median age of the 63 women and 37 men was 32 years (range 18–60) and 27 years (range 19–54), respectively. The median Hemoglobin of the 63 women and 37 men was 11.8 g/dL (range 7.6–14.8) and 14.1 g/dL (range 10.8–16.3), respectively.

As depicted in Figs 1 and 2, compared to the Sysmex XS-1000i laboratory reference, results were most accurate for the HemoCue (bias 0.16, limits of agreement –2.16 to 2.47 g/dL), less accurate for the STAT-Site M^Hgb^ (bias 0.51, limits of agreement –1.98 to 2.99 g/dL), and least accurate for the URIT-12 (bias 2.65, limits of agreement –0.09 to 5.41 g/dL).

**Fig 3:**
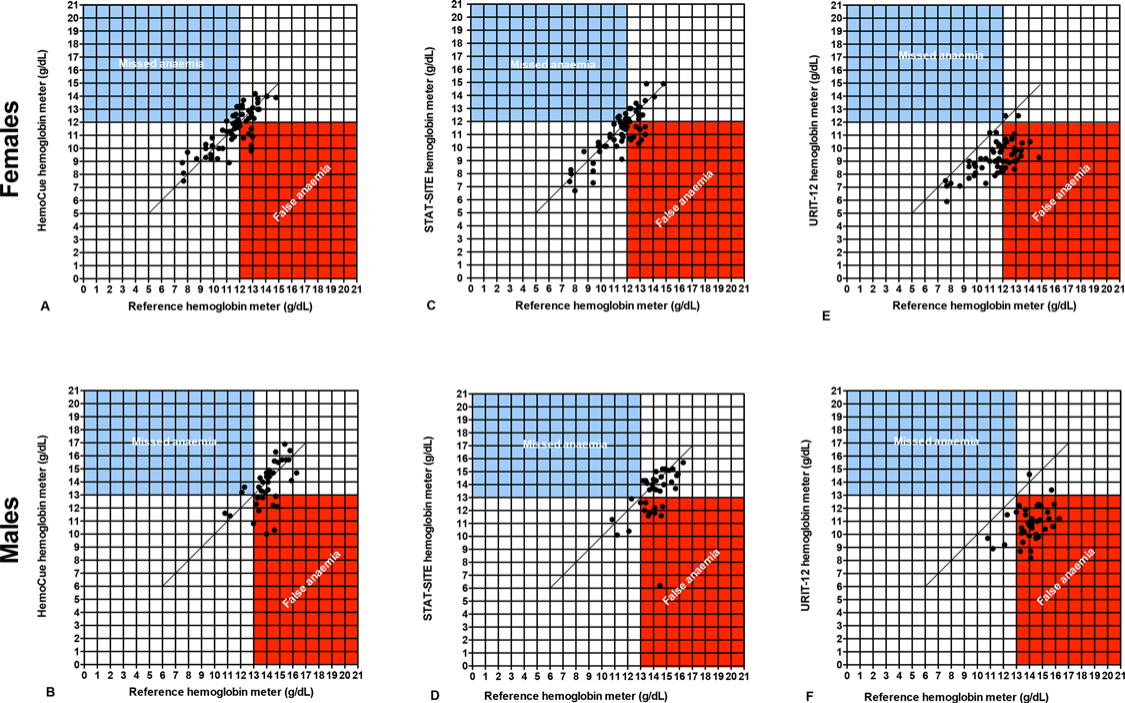
Correlation of the 3 point-of-care assays with values within the dynamic range of the reference hemoglobin meter in 100 samples in a community based clinical setting in phase 2 of the study. Dots in the blue quadrant indicate that the point-of-care assay missed anemia, i.e., misclassified a finger prick sample as non-anemic where the venous blood sample was classified as anemic according to the reference assay in a central laboratory. Dots in the red quadrant indicate that the point-of-care assay misclassified as anemic a sample that was non-anemic according to the reference assay.

The sensitivity and specificity of the point-of-care assays to detect or exclude anemia in a community based clinical setting is depicted in Table 1 and Fig 3. Of note, the sensitivity of the URIT-12 to detect anemia in the community based clinical setting was 100%, but this number must be seen in context of the considerable bias in the results: out of 100 patients, 96 were diagnosed as anemic by the URIT-12. Of those 96 patients diagnosed by URIT-12 as anemic, only 40 (positive predictive value 42%) were truly anemic according to the laboratory reference and the specificity was 7%, with only 4 of 60 non-anemic patients correctly diagnosed as non-anemic by the URIT-12. The clinical consequence of this structural misclassification by the URIT-12 is overdiagnosis of anemia, and overtreatment. The additional consequence in research settings is that many potential study participants will needlessly be excluded from clinical studies, based on the false assumption that they are anemic.

**Table 1:** Phase 2 (community based clinical setting) sensitivity, specificity, and predictive values for the 3 point-of-care tests to detect or exclude anemia.

Females (12 g/dl cut-off)								
Point-of-care tests		reference						
	Outcome	Positive	Negative	Subtotal	sensitivity	specificity	PPV	NPV
HemoCue	Positive	26	8	34	72%	70%	76%	66%
	Negative	10	19	29				
	Total	36	27	63				
Stat-Site	Positive	30	11	41	83%	59%	73%	73%
	Negative	6	16	22				
	Total	36	27	63				
URIT	Positive	36	25	61	100%	7%	59%	100%
	Negative	0	2	2				
	Total	36	27	63				
Males (13 g/dl cut-off)								
Point-of-care tests		reference						
	Outcome	Positive	Negative	Subtotal	sensitivity	specificity	PPV	NPV
HemoCue	Positive	2	10	12	50%	70%	17%	92%
	Negative	2	23	25				
	Total	4	33	37				
Stat-Site	Positive	4	10	14	100%	70%	29%	100%
	Negative	0	23	23				
	Total	4	33	37				
URIT	Positive	4	31	35	100%	6%	11%	100%
	Negative	0	2	2				
	Total	4	33	37				
Females and males (using the respective cut-off values)								
Point-of-care tests		reference						
	Outcome	Positive	Negative	Subtotal	sensitivity	specificity	PPV	NPV
HemoCue	Positive	28	18	46	70%	70%	61%	78%
	Negative	12	42	54				
	Total	40	60	100				
Stat-Site	Positive	34	21	55	85%	65%	62%	87%
	Negative	6	39	45				
	Total	40	60	100				
URIT	Positive	40	56	96	100%	7%	42%	100%
	Negative	0	4	4				
	Total	40	60	100				

Sensitivity = % of patients with anemia, according to the reference laboratory test, that were identified as anemic by the point-of-care test; specificity = % of patients without anemia, according to the reference laboratory test, that were identified as non-anemic by the point-of-care test; PPV, positive predictive value = % of patients that were identified as anemic by the point-of-care test that were confirmed as anemic by the reference laboratory test; NPV, negative predictive value = % of patients that were identified as non-anemic by the point-of-care test that were confirmed as non-anemic by the reference laboratory test.

## Discussion

We found that the diagnostic accuracy of 3 point-of-care hemoglobin meters is different in central laboratory and community based clinic settings, with a trend towards greater variation of results in the community based clinic, for which there are 2 possible explanations: *First*, in the community based clinic setting we compared blood obtained by finger prick for the point-of-care meter with venous blood for the reference laboratory meter. Differences between finger stick and venous hemoglobin measurements have been observed in several studies, with finger stick measurements both underestimating and overestimating hemoglobin levels [10, 11]. *Second*, in phase 2 of the study the point-of-care test was performed by a nurse in a community based clinic, while the central laboratory test was performed by a laboratory technician.

The benefit of our 2 phase approach is that in phase 1 in a central laboratory we could already see the structural bias of the URIT-12, and a 10% invalid results rate for the STAT-Site. Based on the phase 1 results we could have rejected both the STAT-Site and the URIT-12. In phase 2 the structural bias of the URIT-12 was confirmed, but the performance of the Stat-Site was slightly better than during phase 1. This 2 phase approach shows that good performance of a point-of-care test in a central laboratory does not guarantee good performance in a community based clinic setting. Evaluation of a point-of-care test in *only* a community based clinical setting will make it difficult to determine causes of poor performance. Therefore we recommend assessment of the diagnostic accuracy of point-of-care tests in both central laboratory and community based (clinical) settings.

Approval and registration of diagnostics is usually based on central laboratory studies alone. Our study confirms that independent central laboratory and community based evaluations of diagnostic accuracy are important. Appropriate analyses of the data may reveal problems that may not be apparent in the initial registration studies [12, 13]. Several community based evaluations of point-of-care tests [14–18] have revealed problems that do not typically appear in a controlled laboratory environment. Government regulations on the use of diagnostics in South Africa exist but are not very detailed. There seems to be widespread use of point-of-care tests that have not been properly evaluated. Ideally, a diagnostic test should (i) receive approval from regulatory authorities such as the FDA, or CE-marking, and (ii) be evaluated in both central laboratory and community based settings by at least two independent groups, according to STARD guidelines [19–22] (http://www.equator-network.org/reporting-guidelines/stard/), with the results published in the peer reviewed literature.

Another challenge in many African settings is that for many values the clinical laboratory reference intervals derived from Western countries may not be appropriate. Karita et al established reference intervals for routine haematology and biochemistry in healthy adults in Eastern and Southern Africa [23]. Compared to Western reference intervals, the average hemoglobin was lower in several populations. Many otherwise healthy adults would be defined as anaemic, and excluded from clinical studies using inclusion and exclusion criteria based on laboratory reference intervals from other populations [2].

Taken together, the combination of poor regulation, inaccurate tests, and inappropriate reference intervals creates two problems for clinical medicine, and three problems for clinical studies in sub-Saharan Africa. The two problems for clinical medicine are that on the one hand false anemia will lead to costly and unnecessary overtreatment, and on the other hand true anemia will be missed possibly resulting in greater morbidity and associated costs. The three problems for clinical research are that (i) it is difficult to recruit per se due to inappropriate reference intervals, (ii) inaccurate assays will decrease recruitment (false exclusion) and increase premature termination (false inclusion), and (iii) increase the efforts, duration and costs of clinical studies.

How can we benefit from the portability and speed of point-of-care tests, but also accurately determine whether a patient is truly anemic? A practical solution might be an algorithm where point-of-care results within a chosen interval around the cut-off for anemia must be followed by a laboratory reference test. The width of the interval would depend on the accuracy of the point-of-care assay. The patients with hemoglobin values below the interval would be classified as anemic with a high degree of certainty with the point-of-care assay.

Diagnostic accuracy studies are relatively simple, and many laboratories in sub-Saharan Africa perform in-house and/or community based evaluations of new assays, but few publish the results [24, 25]. Diagnostic accuracy studies are good introductions to laboratory and clinical research. Generic protocols can be developed and used, with minor modifications, for different tests. This is a tremendous opportunity to build research capacity, and at the same time address the practical clinical need to assess the diagnostic accuracy of new tests.

## Conclusion

In this study, we have shown that the HemoCue Hb301 is a reasonably accurate point-of-care hemoglobin meter, but the STAT-Site M^Hgb^ and URIT-12 are not. When the assays are performed in a community based setting, none of them can match the performance of a laboratory based reference. The widespread use of an inaccurate point-of-care hemoglobin meter such as the STAT-Site M^Hgb^ and especially the URIT-12 in the public health sector in South Africa is a concern, as it may result in both overtreatment and undertreatment, with ensuing clinical consequences and costs. There is a need for policy guidelines on the validation, approval and quality control of point-of-care assays to (i) improve patient care and (ii) ensure optimal use of scarce resources. The proposed studies can (i) benefit clinical medicine, (ii) benefit clinical research, and (iii) help build research capacity.

## Supporting Information

S1 Fig Correlation of the 3 point-of-care assays with values within the dynamic range of the reference hemoglobin meter in 100 samples in a central laboratory in phase 1 of the study, where all tests were conducted on the same sample. Dots in the blue quadrant indicate that the point-of-care assay missed anemia, i.e., misclassified a sample as non-anemic that was anemic according to the reference. Dots in the red quadrant indicate that the point-of-care assay misclassified as anemic a sample that was non-anemic according to the reference.
(EPS)

S1 Table Phase 1 (central laboratory) sensitivity, specificity, and predictive values for the 3 point-of-care hemoglobin meters to detect or exclude anemia. Sensitivity = % of patients with anemia, according to the reference laboratory test, that were identified as anemic by the point-of-care test; specificity = % of patients without anemia, according to the reference laboratory test, that were identified as non-anemic by the point-of-care test; PPV, positive predictive value = % of patients that were identified as anemic by the point-of-care test that were confirmed as anemic by the reference laboratory test; NPV, negative predictive value = % of patients that were identified as non-anemic by the point-of-care test that were confirmed as non-anemic by the reference laboratory test.
(DOCX)

S2 Table STARD checklist for reporting of studies of diagnostic accuracy *(version January 2003)*. (DOCX)
