# Supplementary material for: Diagnostic Accuracy of the HemoCue Hb 301, STAT-Site MHgb and URIT-12 Point-of-Care Hemoglobin Meters in a Central Laboratory and a Community Based Clinic in Durban, South Africa
Source: PLoS One. 2016 Apr 5;11(4):e0152184. doi: 10.1371/journal.pone.0152184 (PMC4821624; doi:10.1371/journal.pone.0152184)
Supplement: S1 Table — Sensitivity = % of patients with anemia, according to the reference laboratory test, that were identified as anemic by the point-of-care test; specificity = % of patients without anemia, according to the reference laboratory test, that were identified as non-anemic by the point-of-care test; PPV, positive predictive value = % of patients that were identified as anemic by the point-of-care test that were confirmed as anemic by the reference laboratory test; NPV, negative predictive value = % of patients that were identified as non-anemic by the point-of-care test that were confirmed as non-anemic by the reference laboratory test. (DOCX) [file pone.0152184.s002.docx]

**Supplementary table 1.** Phase 1 (central laboratory) sensitivity, specificity, and predictive values for the 3 point-of-care hemoglobin meters to detect or exclude anemia.

| Females (12 g/dl cut-off) | | | | | |  | |  | |  | |  | |  | |  |  |
| --- | --- | --- | --- | --- | --- | --- | --- | --- | --- | --- | --- | --- | --- | --- | --- | --- | --- |
| Point-of-care tests | | | | reference | | | |  | |  | |  | |  | |  |  |
|  | | Outcome | | Positive | | Negative | | Subtotal | |  | | sensitivity | | specificity | | PPV | NPV |
| HemoCue | | Positive | | 20 | | 0 | | 20 | |  | | 91% | | 100% | | 100% | 92% |
|  | | Negative | | 2 | | 23 | | 25 | |  | |  | |  | |  |  |
|  | | Total | | 22 | | 23 | | 45 | |  | |  | |  | |  |  |
|  | |  | |  | |  | |  | |  | |  | |  | |  |  |
| Stat-Site | | Positive | | 20 | | 4 | | 24 | |  | | 100% | | 80% | | 83% | 100% |
|  | | Negative | | 0 | | 16 | | 16 | |  | |  | |  | |  |  |
|  | | Total | | 20 | | 20 | | 40 | |  | | 4 invalid results in 45 samples | | | | |  |
|  | |  | |  | |  | |  | |  | |  | |  | |  |  |
| URIT | | Positive | | 22 | | 21 | | 43 | |  | | 100% | | 9% | | 51% | 100% |
|  | | Negative | | 0 | | 2 | | 2 | |  | |  | |  | |  |  |
|  | | Total | | 22 | | 23 | | 45 | |  | |  | |  | |  |  |
| Males (13 g/dl cut-off) | | | | | |  | |  | |  | |  | |  | |  |  |
| Point-of-care tests | | | | reference | | | |  | |  | |  | |  | |  |  |
|  | | Outcome | | Positive | | Negative | | Subtotal | |  | | sensitivity | | specificity | | PPV | NPV |
| HemoCue | | Positive | | 4 | | 0 | | 4 | |  | | 80% | | 100% | | 100% | 91% |
|  | | Negative | | 1 | | 10 | | 11 | |  | |  | |  | |  |  |
|  | | Total | | 5 | | 10 | | 15 | |  | |  | |  | |  |  |
|  | |  | |  | |  | |  | |  | |  | |  | |  |  |
| Stat-Site | | Positive | | 5 | | 2 | | 7 | |  | | 100% | | 75% | | 71% | 100% |
|  | | Negative | | 0 | | 6 | | 6 | |  | |  | |  | |  |  |
|  | | Total | | 5 | | 8 | | 13 | |  | | 2 invalid results in 15 samples | | | | |  |
|  | |  | |  | |  | |  | |  | |  | |  | |  |  |
| URIT | | Positive | | 5 | | 6 | | 11 | |  | | 100% | | 40% | | 45% | 100% |
|  | | Negative | | 0 | | 4 | | 4 | |  | |  | |  | |  |  |
|  | | Total | | 5 | | 10 | | 15 | |  | |  | |  | |  |  |
| Females and males combined (using the respective cut-off values) | | | | | | | | | | |  | |  | |  | |  |
| Point-of-care tests | | | reference | | | |  | |  | |  | |  | |  | |  |
|  | Outcome | | Positive | | Negative | | Subtotal | |  | | sensitivity | | specificity | | PPV | | NPV |
| HemoCue | Positive | | 24 | | 0 | | 24 | |  | | 89% | | 100% | | 100% | | 92% |
|  | Negative | | 3 | | 33 | | 36 | |  | |  | |  | |  | |  |
|  | Total | | 27 | | 33 | | 60 | |  | |  | |  | |  | |  |
|  |  | |  | |  | |  | |  | |  | |  | |  | |  |
| Stat-Site | Positive | | 25 | | 6 | | 31 | |  | | 100% | | 79% | | 81% | | 100% |
|  | Negative | | 0 | | 22 | | 22 | |  | |  | |  | |  | |  |
|  | Total | | 25 | | 28 | | 53 | |  | | 6 invalid results in 60 samples | | | | | |  |
|  |  | |  | |  | |  | |  | |  | |  | |  | |  |
| URIT | Positive | | 27 | | 27 | | 54 | |  | | 100% | | 18% | | 50% | | 100% |
|  | Negative | | 0 | | 6 | | 6 | |  | |  | |  | |  | |  |
|  | Total | | 27 | | 33 | | 60 | |  | |  | |  | |  | |  |


Sensitivity = % of patients with anemia, according to the reference laboratory test, that were identified as anemic by the point-of-care test; specificity = % of patients without anemia, according to the reference laboratory test, that were identified as non-anemic by the point-of-care test; PPV, positive predictive value = % of patients that were identified as anemic by the point-of-care test that were confirmed as anemic by the reference laboratory test; NPV, negative predictive value = % of patients that were identified as non-anemic by the point-of-care test that were confirmed as non-anemic by the reference laboratory test.
